# Global landscape of mouse and human cytokine transcriptional regulation

**DOI:** 10.1093/nar/gky787

**Published:** 2018-09-03

**Authors:** Sebastian Carrasco Pro, Alvaro Dafonte Imedio, Clarissa Stephanie Santoso, Kok Ann Gan, Jared Allan Sewell, Melissa Martinez, Rebecca Sereda, Shivani Mehta, Juan Ignacio Fuxman Bass

**Affiliations:** 1 Department of Biology, Boston University, Boston, MA 02215, USA; 2 Bioinformatics Program, Boston University, Boston, MA 02215, USA

## Abstract

Cytokines are cell-to-cell signaling proteins that play a central role in immune development, pathogen responses, and diseases. Cytokines are highly regulated at the transcriptional level by combinations of transcription factors (TFs) that recruit cofactors and the transcriptional machinery. Here, we mined through three decades of studies to generate a comprehensive database, CytReg, reporting 843 and 647 interactions between TFs and cytokine genes, in human and mouse respectively. By integrating CytReg with other functional datasets, we determined general principles governing the transcriptional regulation of cytokine genes. In particular, we show a correlation between TF connectivity and immune phenotype and disease, we discuss the balance between tissue-specific and pathogen-activated TFs regulating each cytokine gene, and cooperativity and plasticity in cytokine regulation. We also illustrate the use of our database as a blueprint to predict TF–disease associations and identify potential TF–cytokine regulatory axes in autoimmune diseases. Finally, we discuss research biases in cytokine regulation studies, and use CytReg to predict novel interactions based on co-expression and motif analyses which we further validated experimentally. Overall, this resource provides a framework for the rational design of future cytokine gene regulation studies.

## INTRODUCTION

Cytokines comprise an array of polypeptides that are critical in the development of the immune system and in the regulation of immune and autoimmune responses (1). The published lists of human cytokines range from 132 to 261 genes depending on whether growth factors, hormones, or the receptors of cytokine genes are included (2–4). Here, we focus on 133 cytokine genes, with a primary role in the immune system, shared by different publications.

Cytokine dysregulation is associated with myriad diseases including autoimmune disorders, susceptibility to infections, and cancer (1,5–8). The expression of cytokine genes is primarily regulated at the transcriptional level through a combination of tissue-specific (TS) transcription factors (TFs) that control cytokine expression in different cell lineages, and pathogen- or stress-activated (PSA) TFs that respond to signaling pathways activated by pathogen-derived ligands or endogenous inflammatory mediators (9,10). Although cytokine transcriptional regulation has been studied for more than three decades, including hallmark models of transcriptional regulation such as the IFNB1 enhanceosome (11), we currently lack a comprehensive view of the gene regulatory network (GRN) involved in controlling cytokine gene expression.

Several databases have been generated that annotate protein–DNA interactions (PDIs). InnateDB reports interactions between TFs and immune-related genes retrieved from different databases such as PubMed and IntAct, a subset of which have been manually curated (12). TRRUST reports interactions involving immune and non-immune genes (13), obtained by data mining and curating article abstracts from Pubmed. However, the overlap between these databases is generally low (20% overlap for cytokine genes), suggesting that they may be incomplete and/or may contain misannotated PDIs. This limits our understanding of the combinatorics involved in cytokine transcriptional regulation, especially in terms of the balance between TS and PSA TFs regulating each cytokine gene, the cooperativity and plasticity in cytokine regulation, and the relationship between TF connectivity and immune phenotype/disease.

Here, we mine through three decades of research to generate a comprehensive and user-friendly database, CytReg (http://cytreg.bu.edu), comprising 843 human and 647 mouse interactions between TF and cytokine genes. We analyze this cytokine GRN and integrate it with phenotypic and functional datasets to provide novel insights into the general principles governing cytokine regulation. In particular, we find a correlation between TF connectivity in the cytokine GRN and immune phenotype. We observe that the balance between PSA and TS TFs is shifted towards PSA TFs for interferons and pro-inflammatory cytokines and we provide a model for cooperative and plastic recruitment of cofactors to cytokine promoters. Using this cytokine GRN, we also present a blueprint for further studies of cytokine misregulation in disease and identify novel TF–disease associations. Finally, we discuss biases and the completeness of the literature-derived cytokine GRN, and provide predictions for novel interactions which we validate using enhanced yeast one-hybrid (eY1H) and reporter assays in human cells.

## MATERIALS AND METHODS

### Generation of CytReg

To obtain a comprehensive list of physical and regulatory PDIs between TFs and cytokine genes, we mined the XML files from ∼26 million articles available in Medline on 10 July 2017, using NBCI’s e-utilities python implementation, for studies mentioning a cytokine, a TF, and an experimental assay. Three broad categories of assays (chromatin immunoprecipitation, electrophoretic mobility shift assays, and functional assays), 1431 TFs, and 133 cytokines were considered (Supplementary Table S1). Alternative names for TFs and cytokines were obtained from the HUGO Gene Nomenclature Committee (www.genenames.org) and curated from the literature. Alternative spellings for names that include Greek letters or hyphens were also considered in the data mining.

The resulting 6878 articles, together with 815 articles annotated in databases such as TRRUST (13) and InnateDB (12), were manually curated to determine whether experimental evidence for the PDIs was provided. A spreadsheet was generated containing, for each mined interaction, the TF and cytokine HGNC names, the TF and cytokine names used in the paper, the type of assay, and the PubMed ID of the paper. Curation was performed based on the entire publication, rather than the abstract alone, because in some cases, PDIs reported in the abstract were based on indirect evidence and in other cases many PDIs identified were only reported in the body of the publication or in the figures. In addition to validating or rejecting mined PDIs, curators annotated the species, the functional activity (activating or repressing) if reported, and additional PDIs absent in the mined list but present in the body of the paper. Each PDI was curated by two independent researchers, and disagreements were resolved by a third senior curator. The resulting database contains 1552 PDIs (843 in human, 647 in mouse, and 62 from other species) for which we annotated the assay used and the regulatory activity identified (Supplementary Table S2). To visualize this complex cytokine GRN we developed CytReg (https://cytreg.bu.edu), a web tool where PDIs can be browsed by species, TFs, cytokines, assay types, and TF expression patterns across different cell-types. In addition, links are provided to Uniprot entries (http://www.uniprot.org) for cytokines and TFs, and to PubMed articles for the PDIs.

### Determination of the level of evidence for PDIs

We classified PDIs as high or low evidence of being direct regulatory interactions (Supplementary Table S3). PDIs detected by a functional assay (e.g. reporter assays and TF knockdown) and an assay measuring direct binding (e.g. chromatin immunoprecipitation and *in vitro* binding assays) were classified as high evidence. PDIs detected by only one type of assay were classified as low evidence.

### Determination of the relationship between TF connectivity and gene expression

The median transcript per million (TPM) expression levels in 20 immune cell-types for TFs with different connectivity in the human cytokine GRN was determined based on expression data published by the Blueprint Epigenome Consortium (14) (http://dcc.blueprint-epigenome.eu). In addition, an expression enrichment score in immune tissues compared to non-immune tissues was determined based on data from 32 tissues from the Expression Atlas (https://www.ebi.ac.uk/gxa/experiments/E-MTAB-2836). Briefly, a pseudocount of 1 was added to all the expression data to reduce the noise from low abundant transcripts. Then, the expression of a TF in a tissue was divided by the average expression of the TF across the 32 tissues to obtain an expression enrichment score. Finally, the average enrichment score per TF was determined for the five immune tissues (lymph node, bone marrow, spleen, tonsils, and appendix) and for the remaining 27 non-immune tissues in the dataset.

### Association between TFs and immune phenotypes and diseases

The association between TFs and immune phenotypes was determined based on phenotypes in knockout mice reported by the Mouse Genome Informatics database (www.informatics.jax.org) as of 12 January 2018 (Supplementary Table S4). Thirty different terms including different immune cells, antibody isotypes, cytokines, inflammation and immune tissues were used to determine whether a reported phenotype should be classified as immune-associated.

Association between TFs and immune disorders (including autoimmune diseases and susceptibility to infections) was obtained from the Human Gene Mutation Database 2013 release and from genome-wide association studies (GWAS) downloaded on 27 July 2017 from the NHGRI-EBI Catalog (Supplementary Table S4) (15,16).

### TF enrichment in PDIs with cytokines expressed in different immune cell types

For each TF, we compared the proportion of cytokine targets corresponding to cytokines expressed in a specific immune cell type, to the proportion of the remaining cytokine targets. A proportion comparison test was used to determine a *P*-value and a Benjamini- Hochberg adjusted *P*-value to account for multiple hypothesis testing. TFs with an adjusted *P*-value lower than 0.1 are included in Supplementary Table S5.

### Pathogen/stress-activated and tissue-specific TFs

PSA TFs were determined from the literature based on their ability to be activated or responsive to signaling pathways triggered by pathogen-associated molecular patterns and/or stress signals (e.g. oxidative stress, heat shock, and danger-associated molecular patterns) (Supplementary Table S6). Tissue-specific TFs were determined by calculating a tissue-specificity score (TSPS) based on expression data from 34 different tissues and cells as previously described (17):

\begin{equation*}{\rm TSPS}\ = \ \sum {p_i}.{{\rm log}_2}\left( {\frac{{{p_i}}}{p}} \right)\end{equation*}

where *p_i_* corresponds to the ratio between the expression level in a tissue and the sum of the expression levels across all 34 tissues; and *p* corresponds to the expected ratio under the assumption of equal expression across all tissues. TFs were considered tissue-specific (TS) if their TSPS ≥0.7, a threshold selected based on the bimodal distribution of TSPS across all TFs (Supplementary Table S6). TFs for which a TSPS could not be calculated because of unavailable expression data, were excluded from the analysis.

### Determination of TF inflammatory scores

For each TF, an inflammatory score (IS) was determined as the difference between the percentage of PDIs with canonical pro-inflammatory cytokines (IL1A, IL1B, IL12A, IL12B, IL18, TNF, IFNG, CSF2, CXCL8 and IL6), and the percentage of PDIs with anti-inflammatory cytokines (IL10, IL11, IL13, IL19, IL1RN, IL24, IL37, IL4, IL5, CXCL17, TGFB1, TGFB2 and TGFB3). For TFs with IS ≥ 0.5 or IS ≤ –0.5 we determined the percentage that have a pro- or anti-inflammatory role, or a role in differentiation based of phenotypes in knockout mice (www.informatics.jax.org).

### TF–disease associations

For each disease (asthma, systemic lupus erythematosus, inflammatory bowel disease, type 2 diabetes, rheumatoid arthritis, tuberculosis infection and cytomegalovirus infection) the Expression Atlas (www.ebi.ac.uk) was searched for cytokines upregulated in the disease state, using a cut-off of 2-fold induction (Supplementary Table S7). TFs enriched in regulating the upregulated cytokines were determined from the human cytokine GRN using the Fisher's exact test (Supplementary Table S8). Multiple hypothesis testing was corrected by calculating the Benjamini-Hochberg adjusted *P*-value and using an FDR threshold of 0.1. The resulting TF–disease associations were plotted using a Circos plot (http://mkweb.bcgsc.ca/tableviewer/).

### TF and cytokine association with autoimmune diseases

TFs and cytokines associated with different autoimmune diseases were obtained from the Human Gene Mutation Database 2013 release, and from GWAS downloaded on 27 July 2017 from the NHGRI-EBI Catalog (Supplementary Table S9) (15,16). The union of gene-disease associations between both databases was considered. Crohn's disease and ulcerative colitis were grouped with inflammatory bowel disease. This list includes coding and noncoding variants, and thus variants that affect protein function or expression levels. Of note, this list of gene-disease associations is not comprehensive as it only includes associations identified in genetic studies (i.e. does not consider environmental or epistatic factors that affect cytokine expression). Significance for enrichment of shared autoimmune diseases between interacting TFs and cytokines was determined by comparing to 1000 randomized versions of the human cytokine GRN. Network randomization was performed by edge switching as previously described (18).

### TF–drug associations

TF–drug associations and information regarding drug function were obtained from Drugbank (19). Agonists and activators were grouped as agonists, antagonist and inhibitors were grouped as antagonists. For each cytokine, the number of TFs targetable by agonists or antagonists was determined.

### Prediction of novel PDIs in the human cytokine GRN

To predict novel PDIs in the human cytokine GRN, for each TF, SEEK (20) was used to search for the top 100 genes co-expressed with the known cytokine targets of the selected TF across >5000 expression profiling datasets. Then, for each cytokine within those 100 genes, the presence of binding sites for the selected TF in the cytokine promoter (2 kb upstream of the transcription start site) was determined using the Scan DNA sequence tool in CIS-BP (http://cisbp.ccbr.utoronto.ca/), the PWM-Logodds algorithm, and a stringent threshold of 10 (21). Enrichment for human PDI predictions reported in mouse was determined by calculating an odds ratio and statistical significance was calculated using the Chi-square test. The 1066 predicted interactions were classified according to confidence: high (two or more TF binding sites and evidence of interaction in the mouse cytokine GRN), medium (two or more TF binding sites but absent from the mouse cytokine GRN, or less than two binding sites but presence in the mouse cytokine GRN), and low (one binding site and absent from the mouse cytokine GRN) (Supplementary Table S10).

### Enhanced yeast one-hybrid (eY1H) assays

eY1H assays were used to detect interactions between TFs and cytokine gene promoters (22,23). This method involves two components: a ‘DNA-bait’ such as cytokine gene promoter, and a ‘TF-prey’. The DNA-bait is cloned upstream of two reporter genes (LacZ and HIS3) and both constructs are integrated into the yeast genome (24,25). The DNA-bait strains generated are then mated with yeast strains expressing TFs fused to the yeast Gal4 activation domain (AD), and if the TF binds the regulatory region, the AD moiety activates the reporter genes. Reporter gene activity is measured by the conversion of colorless X-gal to a blue compound, and by the ability of the yeast to grow on media lacking histidine and to overcome the addition of 3-amino-triazole (3AT), a competitive inhibitor of the His3 enzyme. Each interaction was tested in quadruplicate. Yeast DNA-baits corresponding to promoter regions (2 kb upstream of the transcription start site) of cytokine genes were generated as previously described (Supplementary Table S11) (25,26). The promoter regions of CXCL10, CXCL8, CXCL3, CCL4 and CCL20 were screened for REL binding, while promoter regions for IL17A, IL17F, and IL26 were screened for RORC binding. To identify TFs that interact with the promoters of CCL27 and CCL4L2, the CCL27 and CCL4L2 DNA-bait strains were screened against an array of 1086 human TFs (26).

### Motif analysis

Binding of REL, RORC, RBPJ, TFAP2A/B, PPARG, ATF3, EBF1, ZIC1/3, GCM1 and WT1 were predicted using CIS-BP via the Scan DNA sequence tool, using the PWM-LogOdds method and a stringent threshold of 10 (21). Motif analyses were performed on the same 2 kb regions upstream of the transcription start sites used to perform the eY1H assays (Supplementary Table S11).

### Transient transfections and luciferase assays

HEK293T cells were plated in 96-well opaque plates (∼1 × 10^4^ cells/well) 24 h prior to transfection in 100 μl DMEM + 10% FBS + 1% antibiotic-antimycotic 100×. DNA-bait luciferase reporter clones were generated by cloning the cytokine promoter regions upstream of the firefly luciferase into a Gateway compatible vector generated from pGL4.23[luc2/minP] (26). TF-prey clones were generated by Gateway cloning the TFs into a vector derived from pEZY3 (Addgene) to generate fusions with ten copies of the VP16 activation domain (TF-pEZY3-VP160). Cells were transfected with Lipofectamine 3000 (Invitrogen) according to the manufacturer's protocol using 20 ng of the DNA-bait luciferase reporter vector, 80 ng of the TF-pEZY3-VP160 vector, and 10 ng of renilla luciferase control vector. The empty pEZY3-VP160 vector co-transfected with the recombinant firefly luciferase plasmid was used as a negative control. 48 hours after transfection, firefly and renilla luciferase activities were measured using the Dual-Glo Luciferase Assay System (Promega) according to the manufacturer's protocol. Non-transfected cells were used to subtract background luciferase activities, and then firefly luciferase activity were normalized to renilla luciferase activity.

### Code availability

The code used for the data mining in Medline is available at https://github.com/fuxmanlab/cytreg.

### Statistical analyzes

Statistical analyzes were performed using GraphPad Prism Version 7.01, Excel 2016, or VassarStats (http://vassarstats.net). All tests performed were two-tailed tests.

### Software used to generate figures

Box, bar, histogram, and correlation plots were generated using GraphPad Prism Version 7.01. Heatmaps were generated using matrix2png (https://matrix2png.msl.ubc.ca/). Networks were generated using Cytoscape Version 3.2.1 (http://www.cytoscape.org/).

## RESULTS

### Generation of CytReg

**Figure 1.**
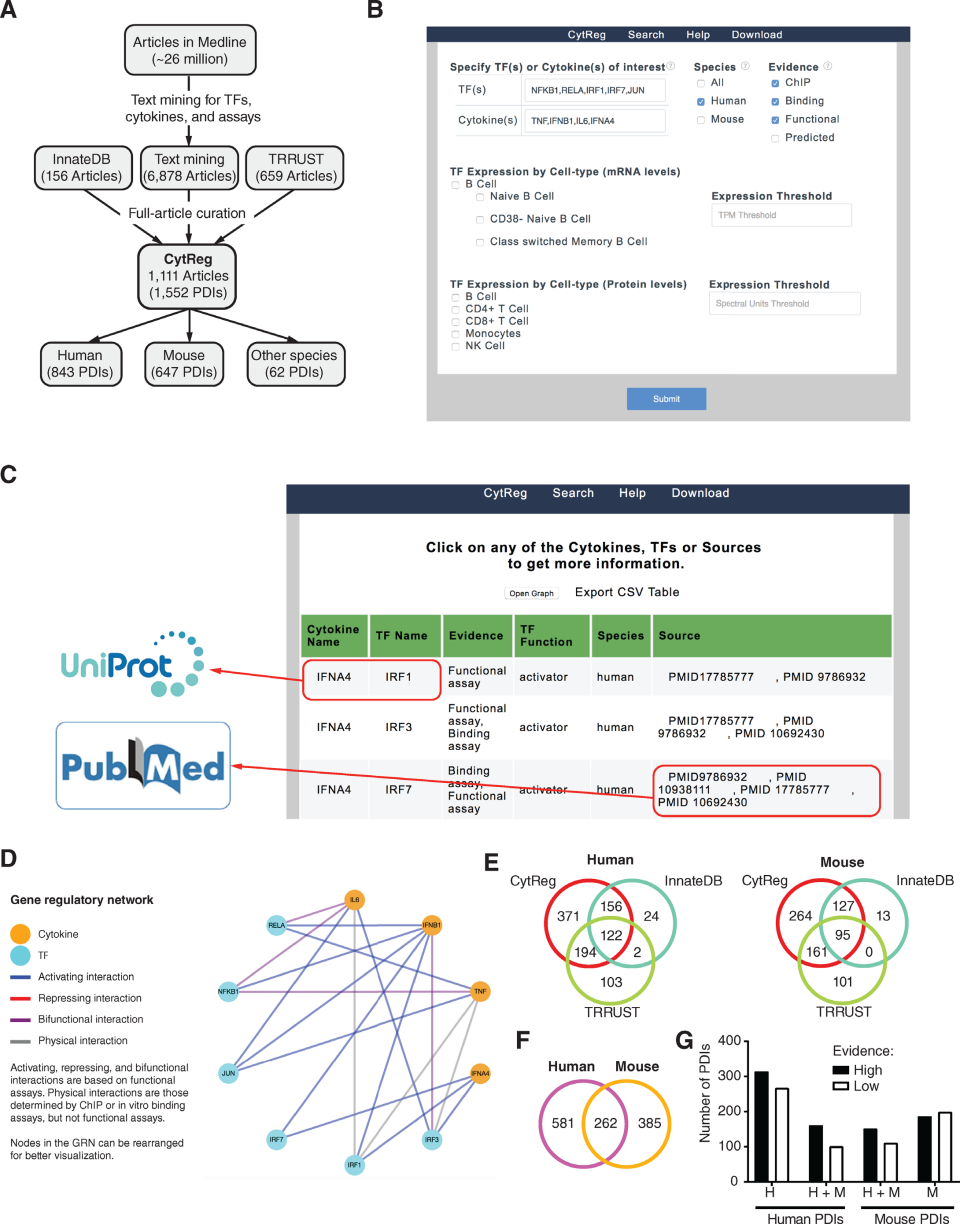
Generation of CytReg. (**A**) Pipeline used for the text mining and article curation to determine literature-based PDIs between TFs and cytokine genes. (**B**) Search page of CytReg where PDIs can be browsed by TF, cytokine, species, assay type, and TF expression levels (mRNA and protein) in different immune cells. (**C**) Results page indicating the interacting cytokines and TFs, the types of assays used to determine the PDIs, whether the interaction is activating or repressing, and the Pubmed IDs of the publications referencing the PDIs. Links are provided to UniProt entries for cytokines and TFs, and to Pubmed for the references. The interactions can be downloaded as a CSV file or visualized as a network graph. (**D**) Network visualization of the selected PDIs. Nodes represent cytokines and TFs, edges represent the type of interaction (activating, repressing, bifunctional, or physical). Nodes can be moved to re-arrange the network. (**E**) Overlap of PDIs in CytReg and those annotated in InnateDB and TRRUST. (**F**) Overlap between mouse and human cytokine GRNs. (**G**) Fraction of PDIs with high evidence of direct regulatory activity (by a functional assay and an *in vitro* or *in vivo* binding assay) or low evidence (by one type of assay).

To obtain a comprehensive cytokine GRN, we systematically mined ∼26 million articles in Medline for studies mentioning at least one of 133 cytokines, one of 1431 TFs, and an experimental assay (Figure 1A and Supplementary Table S1). The resulting 6878 articles, and 815 additional articles referenced in TRRUST (13) and InnateDB (12), were then manually curated to determine whether experimental evidence for the physical and regulatory PDIs was provided. This resulted in a list of 1552 PDIs (843 in human, 647 in mouse and 62 in other species), for which we annotated the assay used and the regulatory activity identified (Figure 1A and Supplementary Table S2). To visualize this GRN we developed a database, CytReg (https://cytreg.bu.edu), where users can browse PDIs by species, TF, cytokine, assay type, and TF expression patterns (Figure 1B). Links are provided to Uniprot entries for TFs and cytokines, and to PubMed articles reporting the PDIs (Figure 1C). Finally, the selected PDIs can be visualized as networks showing the TFs, cytokines, and the types of interactions (activation, repression, or bifunctional) (Figure 1D).

CytReg contains an additional 371 human and 264 mouse PDIs compared to TRRUST and InnateDB (Figure 1E). We also removed 243 PDIs annotated in TRRUST and InnateDB when: (a) the article did not provide direct experimental evidence for the PDI, (b) the TF interacted with the regulatory region of a cytokine receptor rather than that of a cytokine or (c) the cytokine regulated the activation pathway of a TF rather than the TF regulating a cytokine. Altogether, CytReg greatly expands the PDIs annotated in other databases and removes misannotated PDIs.

Although multiple PDIs are shared between human and mouse, 69% of human and 60% of mouse PDIs are species-specific (Figure 1F). This low overlap is not likely related to a lack of confidence in the interactions because a similar proportion of interactions found in one or both species were classified as high confidence based on evidence from functional (e.g. reporters assays and TF knockdowns experiments) and *in vivo* or *in vitro* binding assays (chromatin immunoprecipitation -ChIP- and electrophoretic mobility shift assays –EMSAs, respectively) (Figure 1G and Supplementary Table S3). More likely, this low overlap is related to literature bias and incompleteness of the GRN, or to different modes of regulation between mouse and human as has been previously reported (27). Indeed, we found that PDIs reported early on in one species were more frequently detected in the other species than PDIs reported more recently. For example, 71% of mouse PDIs reported on or before the year 2000 are also reported in human, while only 21% of mouse PDIs reported on or after 2010 are also reported in human. This suggests that literature biases may play an important role in the differences in annotated PDIs between species.

Most interactions were reported by at least two of three types of experimental assays: binding assays (e.g. EMSA and pull down assays), ChIP and functional assays (Supplementary Figure S1A and B). Human PDIs detected by all three types of assays were more frequently also detected in mouse (and *vice versa*) compared to PDIs detected by one or two types of assays (Supplementary Figure S1A and B). The types of assays used to determine PDIs has changed over time, with papers in the 1990s focusing on binding and functional assays while papers in the 2010s focusing on ChIP and functional assays, reflecting the increased awareness of the importance of chromatin context in gene regulation (Supplementary Figure S1C and D).

### Association between TF connectivity and immune phenotype

**Figure 2.**
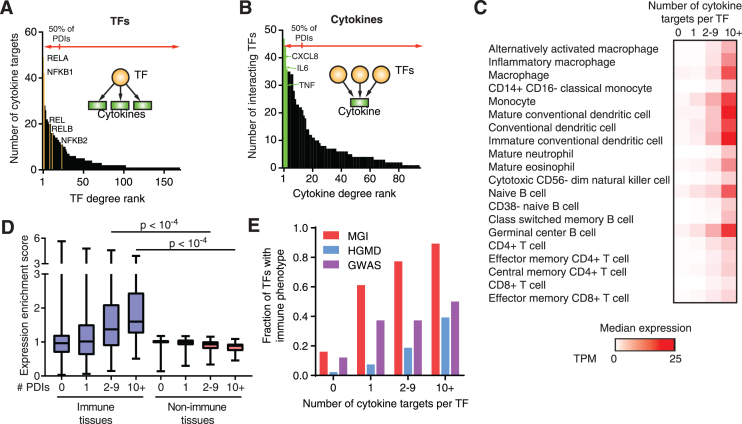
Relationship between TF connectivity and phenotype in the human cytokine GRN. (**A**) Number of cytokine targets per TF (TF degree) in the human cytokine GRN ordered by TF degree rank. (**B**) Number of interacting TFs per cytokine (cytokine degree) in the human cytokine GRN ordered by cytokine degree rank. (**C**) Median expression as transcripts per million (TPM) across human immune cells obtained from the Blueprint Epigenome Consortium for TFs displaying different numbers of cytokine targets. (**D**) Expression enrichment in human immune tissues versus non-immune tissues for TFs with varying numbers of cytokine targets. Each box spans from the first to the third quartile, the horizontal lines inside the boxes indicate the median value and the whiskers indicate minimum and maximum values. Statistical significance determined using two-tailed Wilcoxon matched-pair ranked sign test. (**E**) Fraction of TFs in the human cytokine GRN with annotated immune phenotypes when knocked out in mice (MGI), or associated with immune disorders in the Human Gene Mutation Database (HGMD) or in genome-wide association studies (GWAS) based on the number of cytokine targets.

As observed in other GRNs, a few TFs and cytokines are responsible for most PDIs in the cytokine GRN (Figure 2A and B, and Supplementary Figure S2A and B) (28,29). For example, 12% of the TFs are responsible for >50% of the human PDIs, including different subunits of NF-κB that when combined represent 16% of the PDIs in the human cytokine GRN (Figure 2A). Similarly, 8% of the cytokines, including the highly studied CXCL8, IL6 and TNF, are involved in >50% of the human PDIs (Figure 2B). We obtained similar distributions for the mouse cytokine GRN (Supplementary Figure 2A and B). These lopsided distributions in the number of PDIs can be explained by a more central role of some TFs and cytokines in the GRN, but also by research biases as discussed below.

We found that TFs that interact with multiple cytokine genes show higher expression levels in immune cells (Figure 2C) and higher expression enrichment in immune tissues (such as the spleen, bone marrow, and lymph nodes) compared to TFs that interact with only a few or no cytokine genes (Figure 2D). Further, highly connected TFs are frequently PSA TFs (e.g. 71% of TFs with ten or more cytokine targets are PSA compared to 9% for TFs with one cytokine target) consistent with their function in immune responses. More importantly, highly connected TFs are more frequently associated with immune phenotypes in knockout mouse studies, and with immune disorders as reported in the human gene mutation database (HGMD) and in GWAS compared to low connected TFs (Figure 2E, Supplementary Figure 2C, and Supplementary Table S4) (15,16,30). For example, the highly connected TF IRF5 is associated with multiple autoimmune diseases, including multiple sclerosis and systemic lupus erythematosus (SLE), and leads to low type-I interferon, TNF and IL6 production in knockout mice (15,16,30). Conversely, the low connected TFs HMGA2, NDS2 and HMBOX1, to our knowledge, have not yet been associated with immune phenotypes or diseases. Overall, these observations highlight the association between TF connectivity and disease, consistent with previous findings in a developmental GRN (26).

### Cytokine regulation by different types of TFs

Different cell types express different sets of cytokines in response to pathogen- or cell-mediated cues. For each immune cell type, we determined the TFs enriched in binding/regulating the cytokines expressed in the given cell type (Supplementary Table S5). As expected, several master regulator TFs are enriched, including TBX21 (T-bet) in Th1 cells, GATA3 and STAT6 in Th2 cells, RORC in Th17 cells, and SPI1 (PU.1) and CEBPA in monocytes. Additionally, several PSA TFs, such as RELA/NFKB1, are enriched in Th1 cells, monocytes, myeloid dendritic cells, eosinophils, and neutrophils, consistent with these cells producing pro-inflammatory cytokines upon activation; while IRF1/3/5/7 are enriched in B cells and plasmacytoid dendritic cells, producers of type-I interferons in response to viral pathogens.

**Figure 3.**
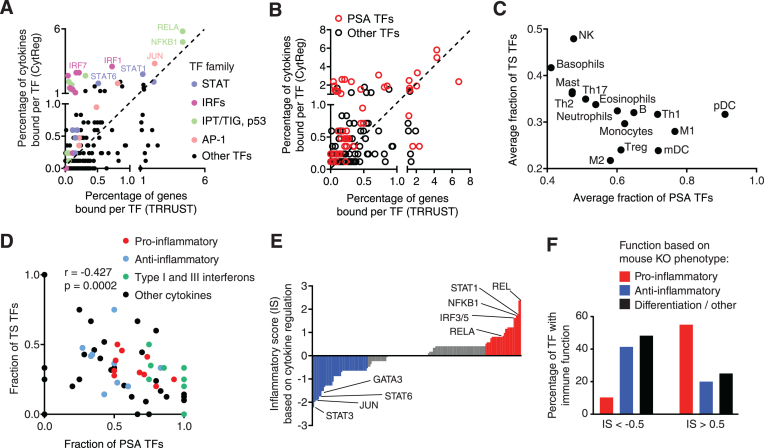
Cytokine regulation by different types of TFs. (A, B) Correlation between the percentage of PDIs involving a TF in the human cytokine GRN versus a global human GRN annotated in TRRUST, for different TF families (**A**) or for pathogen- or stress-activated (PSA) TFs (**B**). (**C**) Average fraction of PSA and tissue-specific (TS) TFs for cytokines expressed in different cell types. (**D**) Fraction of PSA and TS TFs for different classes of cytokines. Correlation determined by Pearson correlation coefficient. (**E**) Inflammatory score (IS) for each TF based on the fraction of PDIs with pro- and anti-inflammatory cytokines. (**F**) Percentage of TFs with pro-inflammatory, anti-inflammatory, and differentiation or other functions based on mouse knockout phenotypes. *P* = 0.009 by Fisher's exact test.

Highly connected TFs in the cytokine GRN usually belong to the Ig-like plexins transcription factor (IPT/TIG/p53 - including NF-κB and NF-AT TFs), activator protein 1 (AP-1), interferon regulatory factor (IRF), and signal transducer and activator of transcription (STAT) families, which are known to play prominent roles in immune cell differentiation and immune responses (31–34). These TF families are highly enriched in the cytokine GRN compared to the GRN reported in TRRUST (13), a literature-derived network not constrained to cytokine genes (Figure 3A and Supplementary Figure S3A and B). Furthermore, most PSA TFs are enriched in the cytokine GRN compared to the GRN reported in TRRUST, consistent with many cytokine genes being upregulated in response to pathogens or stress conditions (Figure 3B).

Cytokines are expressed in a highly tissue- and condition-specific manner. This is achieved by a specific combination of receptors and signaling pathways present in each cell type, and through the cooperation between PSA and TS TFs (31). To study the role of PSA and TS TFs in cytokine regulation, for each cytokine we determined the fraction of TFs that respond to pathogen/stress signals (e.g. NF-κB, AP-1 and IRFs) and the fraction of TS TFs determined based on each TF’s gene expression variability across tissues (Supplementary Table S6). Our analysis revealed that cytokines expressed in plasmacytoid dendritic cells, M1 macrophages, Th1 cells, and myeloid dendritic cells are primarily regulated by PSA TFs, whereas cytokines expressed NK cells, basophils, mast cells, Th2 cells, Th17 cells, and eosinophils are also regulated by several TS TFs (Figure 3C). This is consistent with reports of the former cell types expressing multiple canonical pro-inflammatory cytokines and/or interferons, which are induced by pathogen-associated molecular patterns or danger signals from inflammatory microenvironments. Indeed, further analysis revealed that interferons and pro-inflammatory cytokines are regulated by broadly-expressed PSA TFs, whereas anti-inflammatory cytokines are regulated by both PSA and TS TFs (Figure 3D).

Different TFs have predominantly pro- or anti-inflammatory functions. Thus, for each TF, we determined an inflammatory score (IS) based on the preference of binding to pro- versus anti-inflammatory cytokine gene targets (Figure 3E). TFs with an IS > 0.5 more frequently had a pro-inflammatory function, while TFs with IS < -0.5 more frequently had an anti-inflammatory function based on knockout mouse phenotypes (Figure 3F, *P* = 0.009 by Fisher's exact test). Although the dysregulation of other targets is likely involved, these analyses suggest that the cytokine targets of a TF can be important drivers of immune phenotypes.

### GRN integration with TF-cofactor interactions

**Figure 4.**
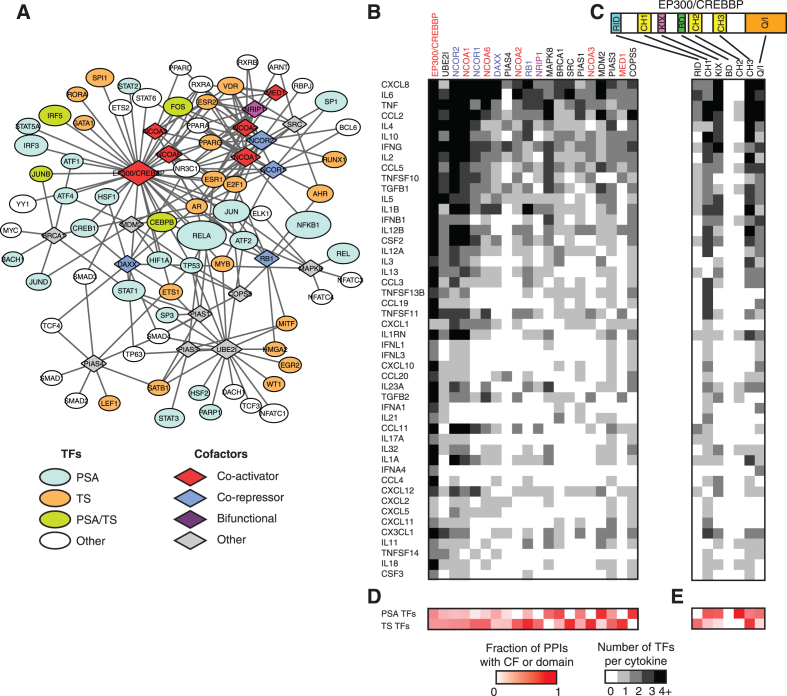
Cooperativity and plasticity in cytokine regulation. (**A**) Protein-protein interaction network from Lit-BM-13 between cofactors and TFs in the human cytokine GRN. Ellipses – TFs, diamonds – cofactors. Node size indicates the number of cytokine targets (for TFs) in the cytokine GRN, and the number of protein-protein interactions with TFs (for cofactors). Only cofactors with five or more protein-protein interactions are shown. (B, C) Number of TFs (shades of grey) interacting with each human cytokine gene that interact with the different cofactors (**B**) or the different domains of EP300/CREBBP (**C**). (D, E) Fraction of cofactor (**D**) or EP300/CREBBP domain (**E**) protein-protein interactions (shades of red) involving PSA or TS TFs. Only cytokines and cofactors with five or more interactions are shown. Co-activators are shown in red font, co-repressors in blue font, and bifunctional cofactors in purple font.

TFs regulate gene expression by recruiting co-activators and co-repressors that interact with the transcriptional machinery or mediator complex, or that covalently modify histones, TFs or methylate DNA (35). Based on literature-derived protein-protein interactions reported in Lit-BM-13 (36), we found that the TFs that bind/regulate cytokine genes interact with numerous cofactors, including multiple co-activators such as EP300, CREBBP and nuclear co-activators 1–3 and 6 (Figure 4A). This is not surprising given that ∼80% of the regulatory PDIs in CytReg are activating and involve potent transcriptional activators such NF-κB and AP-1. Nevertheless, several activating TFs also interact with co-repressors which can inhibit TF function until triggered by signaling pathways (37).

In general, each cofactor interacts with multiple TFs that bind/regulate each cytokine gene (Figure 4B) (36). This may be associated with TF cooperativity to recruit cofactors to regulatory regions as has been reported for the cooperative recruitment of EP300 by RELA, IRFs, JUN and HMGA1 to the IFNB1 enhanceosome (11). Alternatively, cofactor binding to multiple TFs may also be associated with regulatory plasticity by which cofactors can be recruited by different sets of TFs to modulate cytokine gene expression in different cell types or conditions. To evaluate these possibilities, we focused on the histone acetyltansferases EP300/CREBBP, which play key roles in immune regulation and differentiation, and whose protein-protein interactions with TFs have been mapped to their different domains (38,39). We found that, for cytokines for which multiple PDIs have been determined, the set of TFs that bind/regulate that cytokine gene collectively interact with multiple domains of EP300/CREBBP (Figure 4C). This may lead to a cooperative recruitment of EP300/CREBBP to regulatory regions, as has been observed for the IFNB1, TNF, and IL6 genes (11,40,41). This is also consistent with the observation that, even for cytokines with multiple annotated PDIs, the mutation of a single TF binding site or the inhibition of a single TF can lead to a dramatic effect on gene expression (42,43). Interestingly, for each cytokine, several TFs can also interact with the same domain of EP300/CREBBP (Figure 4C). Although this may contribute to a cooperative recruitment of EP300/CREBBP, it may also increase regulatory plasticity in different cell types and/or under different stimuli by allowing different TF combinations to induce cytokine expression. For example, TNF induction by LPS, calcium or viruses all lead to EP300/CREBBP recruitment to the TNF enhanceosome, however, through different sets of TFs (41).

Some cofactors such as MAPK8, BRCA1, MDM2 and COPS5 preferentially interact with PSA TFs, consistent with their reported function in inflammation and stress responses, and associated immune phenotype in knockout mice (Figure 4D) (30). Other cofactors such as NCOR1/2, NCOA1/2/3/6, RB1, NRIP1, SRC and MED1 interact primarily with TS TFs such as nuclear hormone receptors (36). Interestingly, different domains of EP300/CREBBP interact preferentially with PSA or TS TFs: for example, CH1, KIX and Q/I interact mostly with PSA TFs, whereas RID and CH3 interact mostly with TS TFs (Figure 4E). Altogether, this suggests that PSA and TS TFs cooperate in recruiting EP300/CREBBP through different domains to induce cytokine expression under the right stimuli and in the appropriate cell types. In addition, functional redundancy between different PSA TFs may allow for the activation of cytokine expression under different conditions. For example, the PSA TFs HIF1A and NF-κB, both of which interact with the CH1 domain of EP300/CREBBP, can independently induce CXCL8 expression (44). Overall, these findings are consistent with a model that contains aspects of both the enhanceosome (i.e. cooperative TF binding is required for regulatory activity) and billboard (i.e. TFs independently regulate gene expression) models of gene regulation, where only certain combinations of TFs present in particular cells or conditions can induce gene expression (45). Each cytokine, depending on their regulatory flexibility, may be closer to one model or the other.

### The cytokine GRN as a blueprint to study disease

**Figure 5.**
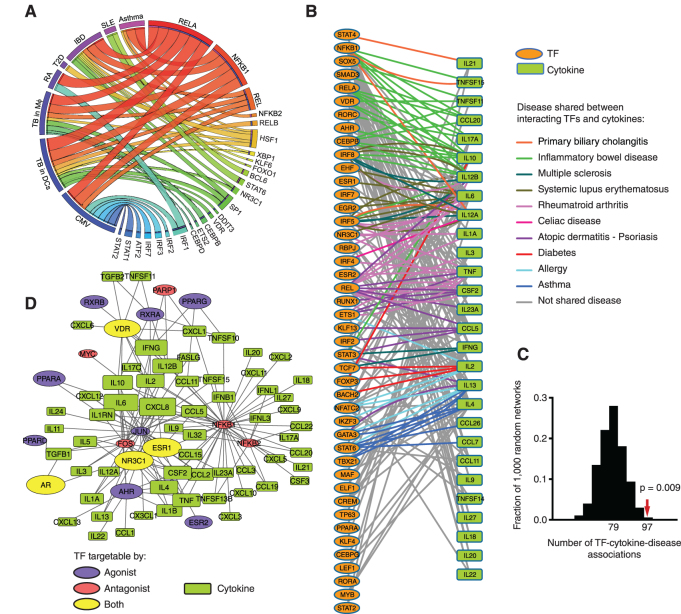
Association of the cytokine GRN with human diseases. (**A**) Circos plot connecting diseases with TFs based on enrichment of the TFs in regulating cytokines upregulated in the indicated disease. Ribbon width is proportional to the percentage of cytokines upregulated in the indicated disease that are regulated by the indicated TF. (**B**) GRN connecting interacting TFs and human cytokine genes associated with autoimmune disorders. Edges connect interacting cytokine-TF pairs. Edge color indicates that the interacting cytokine and TF are associated with the same disease based on HGMD and GWAS. (**C**) The human cytokine GRN was randomized 1000 times by edge switching and the number of TF–cytokine-disease sets in each randomized network was calculated. The number under the histogram peak indicates the average overlap in the randomized networks. The red arrow indicates the observed overlap in the real network. Statistical significance determined based on *z*-score calculation. (**D**) GRN connecting cytokines with TFs that can be targeted by approved drugs. Blue, red, and yellow ovals indicate TFs targetable by agonists, antagonists, or both, respectively. Oval size corresponds to the number of approved drugs targeting a TF. Rectangles indicate cytokine genes. Rectangle size is proportional to the number of drugable TFs per cytokine.

Cytokine expression is widely dysregulated in immune disorders and infection. This is driven by the activation of multiple signaling pathways that result in TF activation leading to the concomitant regulation of target cytokines. To explore these TF–disease relationships, we leveraged the human cytokine GRN to identify TFs enriched in regulating the cytokines overexpressed in different autoimmune diseases, *Mycobacterium tuberculosis* infection, and cytomegalovirus infection (Supplementary Table S7). We identified 46 TF–disease associations between 25 TFs and seven diseases, many of which are known (Figure 5A, and Supplementary Table S8). For example, different subunits of NF-κB were associated with all the diseases evaluated, consistent with the ubiquitous role of NF-κB in inflammation (37). Other TF–disease associations identified were more specific. For instance, IRFs and ATF2 (in addition to NF-κB) were associated with cytomegalovirus infection which is consistent with these TFs being activated by viral pathogens through pattern recognition receptors (46–48). STAT1 and STAT2 were also associated with cytomegalovirus infection, in this case, likely through the activation of signaling pathways driven by the autocrine/paracrine secretion of type-I and type-II interferons induced by IRF and NF-κB activation. In addition, we identified an association between STAT6 and SLE, consistent with STAT6 deficiency being associated with a better prognosis in mouse models of SLE (49,50), and with STAT6 polymorphisms being associated with SLE in humans (51). Further, we found known associations between KLF6, NR3C1, XBP1 and HSF1 with inflammatory bowel disease further validating our analyses (52–55).

More importantly, we found previously uncharacterized TF–disease associations. For example, we identified an association BCL6 and SLE (Figure 5A). A mouse model of SLE (Def6 and SWAP70 double knockout) showed increased BCL6 protein expression (56). However, the role of BCL6 in cytokine dysregulation in SLE has not been established. Our analyses, suggest that the increased BCL6 levels may be associated with increased levels of CCL1/2/7/8/13 observed in SLE (Supplementary Table S7). We also identified a previously uncharacterized association between ETS2 and cytokine upregulation in *M. tuberculosis* infected macrophages (Figure 5A). ETS2 is an activator that is upregulated 5.7-fold (*P* = 3.6 × 10^−7^) in macrophages infected with *M. tuberculosis* for 48 h (E-MEXP-3521). This increased ETS2 expression, together with ETS2 activation through the MAPK pathway (57), may contribute to cytokine upregulation in *M. tuberculosis* infection. Interestingly, the association between ETS2 and *M. tuberculosis* infection would not have been predicted only based on PDIs from InnateDB and TRRUST. Further, using PDIs from these previous databases we only predicted 21 TF–disease associations, most of them included within the 46 associations predicted based on CytReg, while missing multiple known associations such as those between NF-κB subunits and autoimmune diseases (Supplementary Figure S4). Overall, our analyses predicted novel TF–disease associations which are consistent with known TF functions. Further studies are required to determine the mechanisms of action of BCL6 in SLE and ETS2 in *M. tuberculosis* infections.

Mutations in multiple TFs have been associated with immune disorders such as autoimmune diseases (15,16). The role of TFs in autoimmunity is likely related to the dysregulation of immune genes, in particular cytokines, as they play a central role in immune responses and tolerance (7,8). Indeed, mutations in many cytokine genes have been associated with autoimmunity (15,16). We considered the cytokines and TFs that have been associated with autoimmune diseases in GWAS and HGMD, and found that many TF–cytokine gene pairs that interact in the cytokine GRN have been associated with the same autoimmune disease (Figure 5B). For example, we found multiple TF–cytokine pairs associated with inflammatory bowel disease, rheumatoid arthritis, atopic dermatitis/psoriasis, and SLE (Figure 5B and Supplementary Table S9). Overall, the number of TF–cytokine pairs associated with the same autoimmune disease is higher than that determined in randomized networks derived from the human cytokine GRN (Figure 5C). These TF–cytokine pairs identified may constitute different regulatory axes by which TFs lead to the disease. For example, AHR activation is protective in inflammatory bowel disease, partly due to increased IL10 expression (58). Interestingly, the association between AHR, IL10, and inflammatory bowel disease, together with 19 other TF–cytokine-disease associations was absent in predictions based on PDIs from the union of TRRUST and InnateDB. Altogether, the network depicted in Figure 5B constitutes a blueprint to study other regulatory axes in autoimmunity.

Targeting cytokine activity is a widely used therapeutic approach for multiple autoimmune and inflammatory diseases (19,59). However, only ∼15% of cytokines can currently be directly targeted with approved small molecules or specific antibodies, as reported in Drugbank (19). An alternative strategy is to modulate cytokine production by activating or repressing TF regulatory pathways or by using TF agonists or antagonists (19,37,60). Although the use of antibodies is a more specific therapeutic approach to inhibit cytokine activity, antibodies cannot be used in many cases because: (i) approved antibodies blocking cytokine activity are only available for nine cytokines, (ii) a therapeutic strategy may require the concomitant modulation of multiple cytokines, or (iii) a strategy may require the induction of cytokine activity (e.g. the induction of anti-inflammatory cytokines such as IL10) rather than inhibition. In these cases, modulation of cytokine expression by targeting TFs may provide an effective alternative approach.

Many cytokines can potentially be targeted using drugs against their interacting TFs (or the signaling pathways that activate those TFs). Indeed, multiple TF agonists and antagonists have been approved as therapeutics, including 17 TFs with targets in the human cytokine GRN (Figure 5D). Combined, these TFs, which include nuclear hormone receptors, NF-κB and AP-1, can potentially target 59 cytokine genes, most of which are dysregulated in disease (Supplementary Table S7). Targeting these TFs can increase or decrease cytokine expression depending on the TF regulatory function and on the drug's agonist or antagonist activity. For example, IL10 expression can be induced using AHR agonists as a protective mechanism in inflammatory bowel disease, or repressed by an endogenous VDR agonist (calcitriol) during pregnancy to enhance responses to microbial infections (58,61). Ultimately, multiple factors need to be considered including the off-target effect of the drugs, the number of other genes whose expression may be affected by targeting a particular TF, and how the modulation of TF activity may propagate to other immune and non-immune functions.

### Completeness of the cytokine GRN

**Figure 6.**
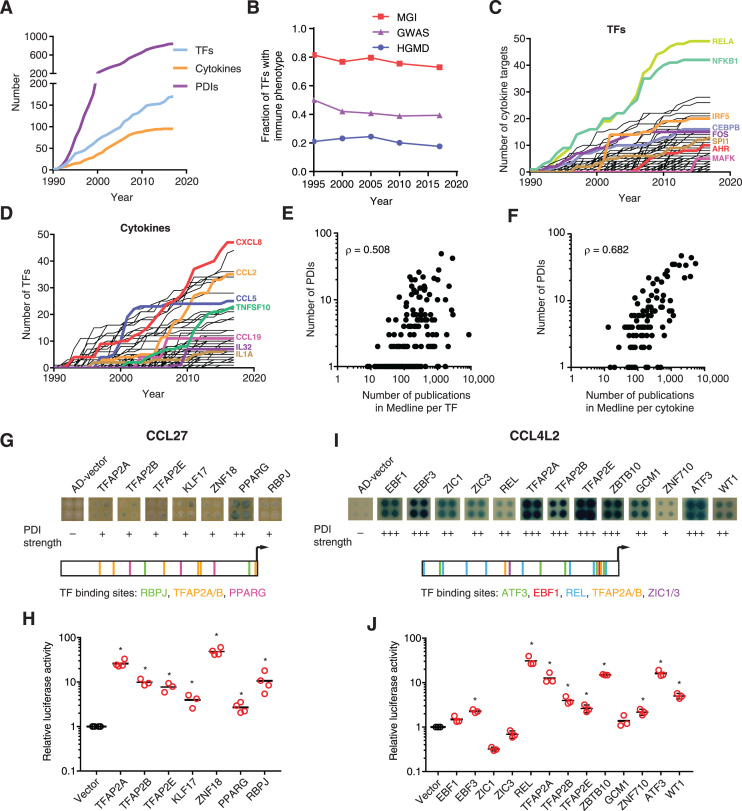
Completeness of the human cytokine GRN. (**A**) Number of annotated PDIs, TFs, and cytokines in the human cytokine GRN over time. (**B**) Fraction of TFs in the human cytokine GRN with annotated immune phenotypes when knocked out in mice (MGI) or associated to immune disorders in genome-wide association studies (GWAS) and in the Human Gene Mutation Database (HGMD) over time. (C, D) Number of PDIs per TF (**C**) or per cytokine (**D**) in the human cytokine GRN over time. (E, F) Correlation between the number of PDIs in the human cytokine GRN and the number of publications per TF (**E**) or per cytokine (**F**) reported in Medline. (G, I) PDIs with the promoters of CCL27 (**G**) or CCL4L2 (**I**) were analyzed by eY1H assays. Each interaction was tested in quadruplicate. The qualitative strength of PDIs detected by eY1H compared to AD-vector control are indicated as –, +, ++ and +++ corresponding to no, weak, medium, and strong interaction, respectively. Motif location for the indicated TFs in the promoters of CCL27 and CCL4L2 are shown. (H, J) Luciferase assays to validate interactions between the promoters of CCL27 (**H**) or CCL4L2 (**J**) and the indicated TFs. HEK293T cells were co-transfected with reporter plasmids containing the cytokine promoter region (2 kb) cloned upstream of the firefly luciferase reporter gene, and expression vectors for the indicated TFs (fused to the activation domain VP160). After 48 h, cells were harvested and luciferase assays were performed. Relative luciferase activity is plotted as fold change compared to cells co-transfected with the vector control (1.0). Experiments were performed 3–4 times in three replicates. Individual data points represent the average of the three replicates, the average of all experiments is indicated by the black line. *P < 0.05 by one-tailed Student's t-test with Benjamini-Hochberg correction.

Although great progress has been made in the last three decades identifying novel PDIs, the cytokine GRN is far from complete. Indeed, we observed that the size of the cytokine GRN and the number of TFs involved have increased at a constant rate suggesting that novel PDIs remain to be identified (Figure 6A and Supplementary Figure S5A). Importantly, the fraction of TFs that have been incorporated into the cytokine GRN that are associated with immune phenotypes or diseases has remained constant suggesting that the GRN continues to grow towards immune-relevant interactions (Figure 6B and Supplementary Figure S5B).

Future growth of the cytokine GRN is not expected to be uniform for all TFs and cytokines. Indeed, the number of PDIs seems to have saturated for some TFs such as RELA, NFKB1, and FOS, while other TFs such as SPI1 and MAFK do not show signs of saturation (Figure 6C and Supplementary Figure S5C). The number of PDIs for some well-studied cytokines such as CCL5 have also plateaued, while new PDIs are still being identified for other cytokines such as human CXCL8 and CCL2 or mouse IL4 (Figure 6D and Supplementary Figure S5D).

We also observed a bias towards highly studied TFs and cytokines as we detected a strong correlation between the number of publications in Medline associated with a cytokine or TF and the number of PDIs in the cytokine GRN (Figure 6E and F; and Supplementary Figure S5E and F). An argument can be made that highly connected TFs have more pleiotropic functions and thus, are more frequently studied. However, more than 200 TFs absent in the cytokine GRN lead to an immune phenotype when knocked out in mice, many of which are associated with alterations in cytokine expression (Supplementary Table S4) (30). This suggests that many TFs are absent from the cytokine GRN and that many PDIs involving infrequently studied TFs are missing.

Similarly, highly studied cytokines are involved in more PDIs (Figure 6F and Supplementary Figure S5F). Although we cannot rule out the possibility that highly studied cytokines have more pleiotropic roles and are regulated by different TFs in different cells and conditions, this alone cannot explain that there are no PDIs reported for 30% of the cytokines. Further, if there is a strong selective pressure to have multiple modes of regulation for certain cytokines, we would expect the mouse and human cytokine orthologs to be regulated by a similar number of TFs, but this is frequently not the case (Supplementary Figure S5G and H). What is more likely is that highly studied cytokines such as TNF and CXCL8 have more PDIs because they have been studied in more cell types and conditions. To test this hypothesis, we performed eY1H assays to evaluate the binding of 1,086 human TFs to the promoters of CCL27 and CCL4L2, two under-studied cytokines absent from the GRN (Figure 6G and I). We detected seven interactions with the CCL27 promoter involving TFAP2A/B/E, KLF7, ZNF18, PPARG, and RBPJ (Figure 6G). Motif analyses for TFs with available position weight matrices (TFAP2A/B, PPARG and RBPJ) identified multiple TF binding sites in the CCL27 promoter. We evaluated the seven eY1H interactions by luciferase assays in HEK293T cells, all of which were validated (Figure 6H). Of note, TF ZNF18, which is widely expressed in immune cells, is also absent from CytReg showing that novel TFs in the cytokine GRN remain to be identified. We also detected 13 TF interactions with the promoter of CCL4L2 using eY1H assays (Figure 6I). Multiple TF binding sites were found in the promoter of CCL4L2 for most of the TFs for which a position weight matrix was available. We tested the 13 eY1H interactions by luciferase assays in HEK293T cells, nine of which validated (Figure 6J). Interestingly, ATF3 is known to regulate CCL4, a close paralog of CCL4L2 (62). Further, CCL4L2 is produced by multiple cell types including monocytes, B cells, T cells, fibroblasts, endothelial, and epithelial cells, while ATF3, EBF3, REL, ZBTB10, ZNF710, WT1, TFAP2A and TFAP2E are also expressed in one or more of these cell types (63). Overall, this shows that novel interactions can be detected for cytokines and TFs that have been poorly characterized.

### Prediction of novel PDIs in the cytokine GRN

**Figure 7.**
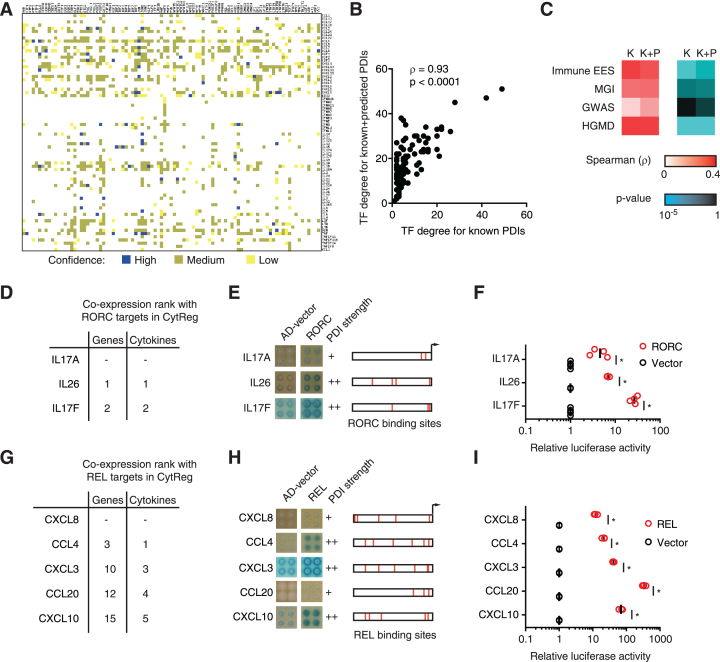
Prediction of novel PDIs in the human cytokine GRN. (**A**) Novel PDI predictions based on co-expression between cytokines and known cytokine targets of each TF (determined using the SEEK database), and motifs analysis. Prediction confidence, as defined in the methods section, is shown. (**B**) Correlation between the number of cytokine targets (TF degree) for known PDIs and known + predicted PDIs. Correlation determined by Spearman's rank correlation coefficient. (**C**) Correlation between TF degree for known (K) or known + predicted (K+P) PDIs and expression enrichment score (EES) in immune tissues, mouse immune phenotype (MGI), and human immune disorders in GWAS and HGMD. Correlation and significance determined by Spearman's rank correlation coefficient. (D, G) Top predicted cytokine targets of RORC (**D**) and REL (**G**). The co-expression rank among all genes and among cytokines is shown. CXCL8 is a known target of REL, while IL17A is a known target of RORC. (E, H) Enhanced yeast one-hybrid assays testing PDIs between the indicated human cytokine promoters and RORC (**E**) and REL (**H**). AD-vector corresponds to empty vector. The qualitative strength of PDIs compared to AD-vector control are indicated as –, +, ++ and +++ corresponding to no, weak, medium, and strong interaction, respectively. REL and RORC binding sites are indicated in red for each 2 kb promoter region. (F, I) Luciferase assays in HEK293T cells co-transfected with reporter plasmids containing the indicated cytokine promoter region (2 kb) cloned upstream of the firefly luciferase reporter gene, and expression vectors for RORC (**F**) or REL (**I**) (fused to the activation domain VP160). After 48 h, cells were harvested and luciferase assays were performed. Relative luciferase activity is plotted as fold change compared to cells co-transfected with the vector control (1.0). Experiments were performed 3–4 times in three replicates. Individual data points represent the average of the three replicates, the average of all experiments is indicated by the black line. *P < 0.05 by one-tailed Student's *t*-test with Benjamini–Hochberg correction.

To predict novel PDIs in the human cytokine GRN, we leveraged the observation that co-expressed genes tend to share interactions with similar TFs (64,65). Thus, for each TF with at least two PDIs in the human cytokine GRN, we searched for other cytokines co-expressed with the known target cytokines across >5000 expression profiling datasets using SEEK (20). Potential targets were then filtered by the presence of the corresponding TF binding site in the promoter region (2 kb upstream of the transcription start site) determined using CIS-BP (21). The 1066 predicted PDIs were enriched in orthologous interactions detected in mouse but absent from the human cytokine GRN (OR = 4.43, *P* < 10^−20^ by Chi-square test). Predictions were classified as high, medium, or low confidence based on the number of TF binding sites for the corresponding TF and the presence of the interaction in mouse (Figure 7A; Supplementary Table S10). As expected, there is a strong correlation between the TF degree for known and for known plus predicted interactions, although this correlation is not perfect (Figure 7B). Importantly, adding the predicted interactions, maintained or even improved the correlation between TF degree and expression enrichment in immune tissues, presence of immune phenotype in mouse, and association with immune disorders in GWAS and HGMD (Figure 7C). Overall, this suggests that our predictions are enriched in functional PDIs.

Using this platform, we predicted IL26 and IL17F to be novel potential targets of RORC, whose RORγt isoform is a master regulator of Th17 cell differentiation and function (Figure 7D) (66). The interaction between RORC and IL17F, a paralog of the known RORC target IL17A, was reported in mouse (67) but, to our knowledge, not in human. IL26 is a key cytokine involved in immune cell priming, antibacterial immunity, and autoimmune diseases produced by RORγt expressing Th17 cells, but not previously shown to be directly regulated by RORγt (68,69). We validated these two novel predicted PDIs using eY1H assays, motif analyses, and luciferase assays in HEK293T cells showing even stronger activity than the well-known RORC-IL17A interaction (Figure 7E and F). Overall, this suggests that RORC directly regulates multiple Th17 cytokines.

Using a similar approach, we found that CCL4, CXCL3, CCL20 and CXCL10 are among the most highly correlated cytokines to the known targets of the well-studied TF REL, and that their promoters have multiple binding sites for REL (Figure 7G and H). Interestingly, these cytokines are known to be regulated by other subunits of NF-κB but, to our knowledge, not by REL (Supplementary Table S2). We validated these predicted interactions using eY1H assays and luciferase assays in HEK293T cells (Figure 7H and I). Interestingly, these four novel targets of REL, a TF associated with autoimmune disorders, are also associated with and/or upregulated in autoimmune disorders (Supplementary Table S7) (70–74). Overall, this shows that by integrating the PDIs annotated in CytReg with co-expression data we can expand the current cytokine GRN. Additionally, our predictions provide a blueprint for further studies in cytokine regulation.

## DISCUSSION

In the present study, we mined ∼26 million articles in Medline, of which we curated >7000 articles, to generate comprehensive mouse and human cytokine GRNs comprising 843 and 647 PDIs, respectively. We created a user-friendly database (https://cytreg.bu.edu) where PDIs can be easily browsed by TF, cytokine, species, assay type, and TF expression patterns, and visualized as networks. Overall, CytReg is 2- to 3-fold more complete than other databases such as InnateDB and TRRUST (12,13). Using this comprehensive database, we were able to obtain novel insights into the principles involved in cytokine regulation, perform comparative analyses between mouse and human GRNs, and make functional predictions which were not previously possible with other databases.

By analyzing the cytokine GRN, we found that highly connected TFs are more highly expressed in immune cells and more frequently associated with immune phenotypes and diseases compared to low connected TFs. This is consistent with previous reports correlating network connectivity and phenotype, both in protein-protein and protein–DNA interaction networks (26,29,75). Interestingly, we found that this correlation is specific to immune diseases as TFs associated with non-immune diseases do not display a high connectivity in the cytokine GRN (not shown). Overall, this suggests that the link between TF connectivity and phenotype may be a local feature of GRNs where connectivity to functionally related targets, rather than the entire GRN, dictates the type of phenotypes or diseases a TF is associated with. For example, REL which is highly connected in CytReg, but not in TRRUST, has been associated with rheumatoid arthritis, psoriasis, and Hodgkin's lymphoma but not with diseases unrelated to the immune system (15).

Our analysis of the combinatorics of the TFs that regulate each cytokine gene illustrates the complexity in cytokine transcriptional regulation. We observed that pro- and anti-inflammatory cytokines are regulated by a different balance between PSA and TS TFs, but ultimately a combination of both types of TFs may be required for cofactor recruitment to induce cytokine expression in the appropriate cells and conditions. This cooperativity between PSA and TS TFs, together with cell type specific expression patterns of surface receptors and signaling molecules, may ultimately be responsible for the tight control of cytokine expression in immune responses. The cooperative relationship between TFs may also explain the deleterious effects of several disease-associated single nucleotide variants (SNVs) and engineered mutations in the promoters and enhancers of cytokine genes, as affecting the binding of a single TF may result in the loss of cooperativity and lead to gene misregulation (43,76,77). For example, using massively parallel reporter assays it was recently shown that ∼60% of all possible substitutions in the core 44 nt of the IFNB1 enhanceosome altered its activity in virus-infected cells (43). Remarkably, most of the substitutions that did not affect activity were located outside of known TF binding sites or led to an alternative binding site for the same TF.

Our analyses also suggest a potential plasticity between TFs in cofactor recruitment, given that frequently multiple TFs that regulate a cytokine gene can interact with the same domain of EP300/CREBBP. Fine-mapping TF interactions with protein domains of other cofactors will indicate whether this is a unique feature of EP300/CREBBP. Further, a comprehensive functional characterization of different substitutions in cytokine promoters may determine whether the substitutions that affect the binding of potentially redundant TFs are generally more benign than those affecting the binding of cooperative TFs. However, the converse can also be true as this plasticity may be required for proper cytokine expression in different cell types and conditions.

CytReg is the most comprehensive cytokine GRN to-date, significantly increasing the number of annotated PDIs compared to previous databases, yet CytReg is not fully complete. First, articles that do not mention interactions within the information available in Medline will be missed and will not have been curated. Second, CytReg is incomplete because multiple PDIs remain to be evaluated and characterized. Indeed, by performing eY1H and luciferase reporter assays, we found interactions involving cytokines (CCL27 and CCL4L2) and TFs (e.g. ZNF18, ZBTB10, KLF17, EBF3 and ZNF710) that are absent from CytReg. Further, by leveraging CytReg, co-expression data, and motif analyses we predicted 1,066 PDIs in the human cytokine GRN, a subset of which we validated by eY1H and luciferase assays. Third, in addition to missing PDIs in the cytokine GRN, individuals may carry genomic variants in noncoding regulatory regions of cytokine genes or in TF coding sequences that lead to different TF–cytokine interactions. Indeed, several disease-associated SNVs have been identified in the promoters of cytokine genes that result in the gain or loss of PDIs that may be absent in CytReg (26,78–80). For example, a SNV in the proximal promoter of CCL5 that is associated with atopic dermatitis leads to a gain of PDI with GATA2 (26,78). Finally, CytReg catalogues PDIs as binary interactions between TFs and cytokine genes. However, the number of binding sites for each TF, their strength, spacing, and orientation are key for appropriate gene expression (45,81). With a few exceptions (e.g. the IFNB1 and the TNF enhanceosomes), this regulatory logic is currently unknown, and thus cannot be annotated (11,41). Ultimately, the integration of different high-throughput and unbiased approaches, population-wide studies of regulatory variation, and in-depth functional characterizations of the regulatory logic will lead to a more comprehensive picture of cytokine regulation in different cell types, conditions, and individuals.

## Supplementary Material

Supplementary Data
