# Supplementary material for: Global landscape of mouse and human cytokine transcriptional regulation
Source: Nucleic Acids Res. 2018 Sep 3;46(18):9321–37. doi: 10.1093/nar/gky787 (PMC6182173; doi:10.1093/nar/gky787)
Supplement: Supplementary Data [file gky787_supplemental_files.zip › Supplementary information.pdf]

SUPPLEMENTARY INFORMATION

Supplementary Figure Legends

Supplementary Figure S1. Distribution of experimental methods used to determine PDIs. (**A, B**) Number of PDIs in the human (**A**) and mouse (**B**) cytokine GRNs per assay type and the number of PDIs annotated in the mouse and human GRNs, respectively. Filled circles – PDIs involving the assay. (**C, D**) Number of PDIs in the human (**C**) and mouse (**D**) cytokine GRNs per assay type over time.

Supplementary Figure S2. Relationship between TF connectivity and phenotype in the mouse cytokine GRN. (**A**) Number of cytokine targets per TF (TF degree) in the mouse cytokine GRN ordered by TF degree rank. (**B**) Number of interacting TFs per cytokine (cytokine degree) in the mouse cytokine GRN ordered by cytokine degree rank. (**C**) Fraction of TFs in the mouse cytokine GRN with annotated immune phenotypes when knocked out in mice (MGI), or associated with immune disorders in the Human Gene Mutation Database (HGMD) or in genome-wide association studies (GWAS) based on the number of cytokine targets.

Supplementary Figure S3. TF families present in the mouse and human cytokine GRNs. (**A**) Correlation between the percentage of PDIs involving a TF in the mouse cytokine GRN versus a global mouse GRN annotated in TRRUST. (**B**) Distribution of TF families

in the human and mouse cytokine GRNs compared to those annotated in the TRRUST database.

Supplementary Figure S4. Circos plot connecting diseases with TFs based on enrichment of the TFs in regulating cytokines upregulated in the indicated disease. Ribbon width is proportional to the percentage of cytokines upregulated in the indicated disease that are regulated by the indicated TF. The left plot is based on PDIs from the union of TRRUST and InnateDB, the right plot is based on PDIs from CytReg (as in **Figure 5A**).

Supplementary Figure S5. Completeness of the mouse cytokine GRN. (**A**) Number of annotated PDIs, TFs, and cytokines in the mouse cytokine GRN over time. (**B**) Fraction of TFs in the mouse cytokine GRN with annotated immune phenotypes when knocked out in mice (MGI) or associated to immune disorders in genome-wide association studies (GWAS) and in the Human Gene Mutation Database (HGMD) over time. (**C, D**) Number of PDIs per TF (**C**) or per cytokine (**D**) in the mouse cytokine GRN over time. (**E, F**) Correlation between the number of PDIs in the mouse cytokine GRN and the number of publications per TF (**E**) or per cytokine (**F**) reported in Medline. Correlation determined by Spearman's rank correlation coefficient. (**G, H**) Correlation between the number of PDIs per TF (out degree) (**G**) or per cytokine (in degree) (**H**) in the human and mouse cytokine GRNs.

Supplementary Table Legends

Supplementary Table S1. TFs, cytokines, and assays used in data mining. TF and cytokine official and alternative names used for mining in Medline. Experimental assays used in the data mining are also indicated.

Supplementary Table S2. List of PDIs in CytReg. List of PDIs between TFs and cytokine genes, assay types, species, activating/repressing interactions, PubMed IDs referencing the PDIs, and year of publication.

Supplementary Table S3. Evidence for direct regulatory PDIs. Evidence for direct regulatory interactions for human and mouse PDIs. High – evidence by functional assay and by ChIP/binding assay, Low – evidence by functional assay or by ChIP/binding assay but not both.

Supplementary Table S4. TF immune phenotypes. Immune phenotypes from knockout mice obtained from the Mouse Genome Informatics (MGI) database, and human diseases obtained from the Human Gene Mutation Database (HGMD) and from GWAS associated with different TFs. 1 = present, 0 = absent. The TF out degree in the human and mouse cytokine GRNs is indicated.

Supplementary Table S5. TFs enriched in PDIs with cytokines expressed in different immune cell types. TFs enriched, p-values, and Benjamini-Hochberg (BH) adjusted p-values are indicated.

Supplementary Table S6. PSA and TS TFs classification. PSA and TS TF classification for TFs in human cytokine GRN. TSPS = tissue-specificity score. 1 = true, 0 = false.

Supplementary Table S7. List of cytokines dysregulated in different diseases obtained from the Expression Atlas (www.ebi.ac.uk). The disease, cytokine, experiment accession ID, sample comparison, fold change in expression, and adjusted p-value are indicated.

Supplementary Table S8. List of TF-disease associations. The number of cytokine targets upregulated in each disease is indicated for each TF, as well as the fold-enrichment, p-value, and adjusted p-value.

Supplementary Table S9. TF and cytokine association with autoimmune diseases. List of TFs and cytokines associated with different autoimmune diseases obtained from the HGMD and GWAS. Pairs of TFs and cytokines genes involved in PDIs that share associated autoimmune disorders are indicated.

Supplementary Table S10. Predictions of novel PDIs in the human cytokine GRN. Predicted interactions based of coexpression with known TF targets, number of motifs in

the cytokine promoter for the indicated TF, and presence of the interaction in the mouse cytokine GRN are indicated.

Supplementary Table S11. Cytokine promoters tested by eY1H and luciferase assays. Cytokine genes, promoter genomic coordinates, promoter sequences, and primers used for cloning corresponding to the cytokines promoters tested by eY1H assays, luciferase assays, and motif analyses.
