# Supplementary figures and images for: Global landscape of mouse and human cytokine transcriptional regulation
Source: Nucleic Acids Res. 2018 Sep 3;46(18):9321–37. doi: 10.1093/nar/gky787 (PMC6182173; doi:10.1093/nar/gky787)

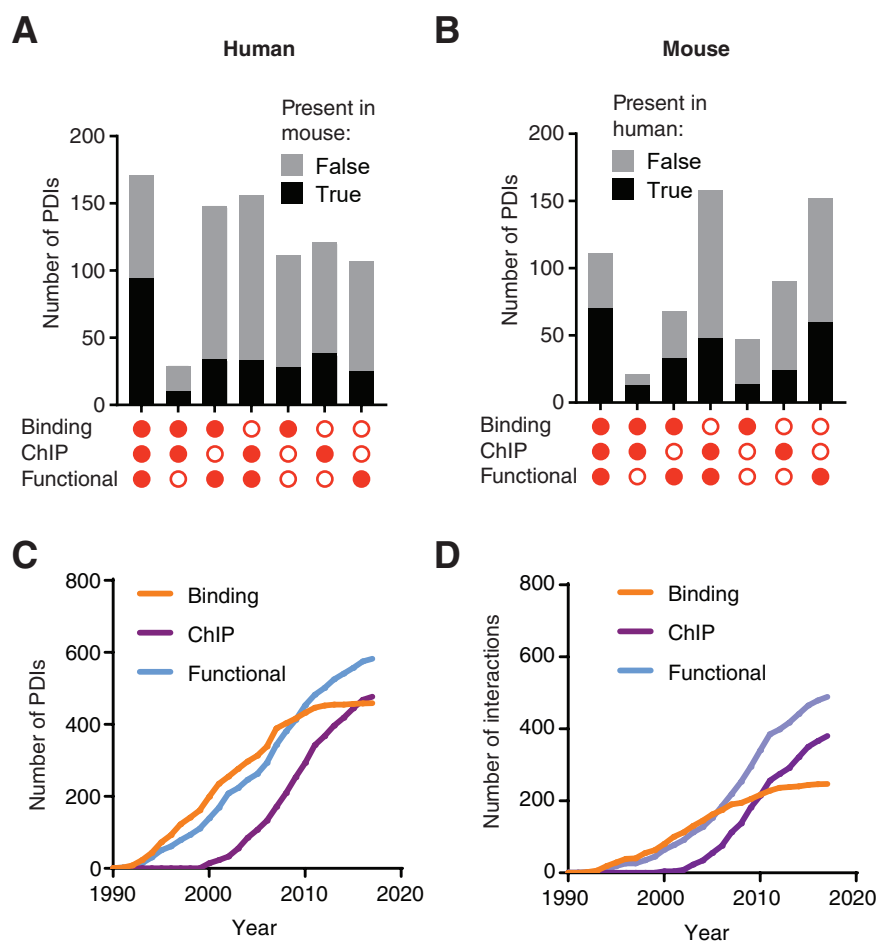

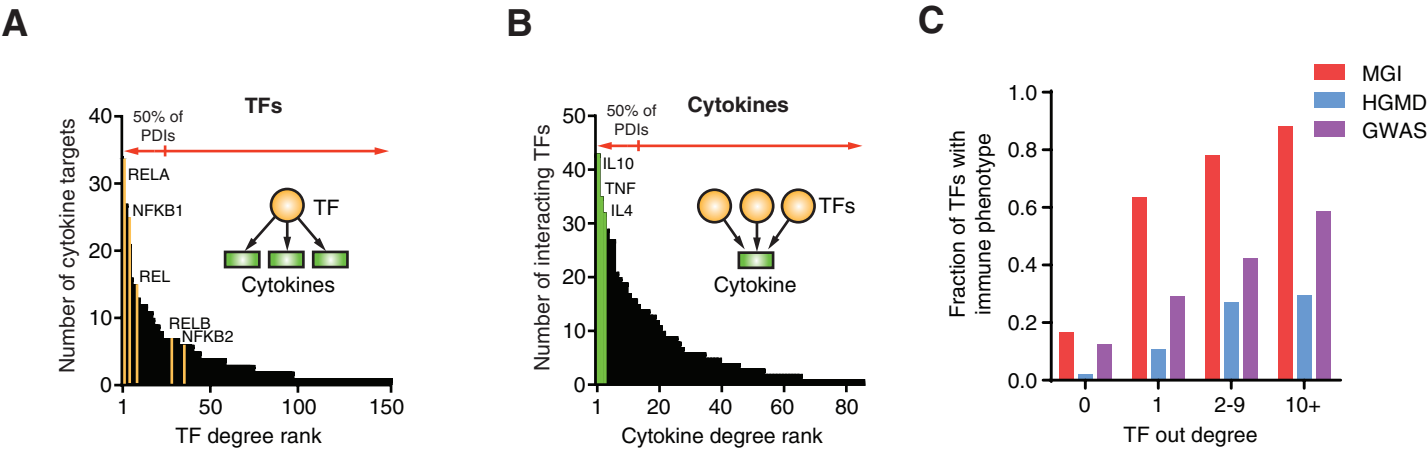

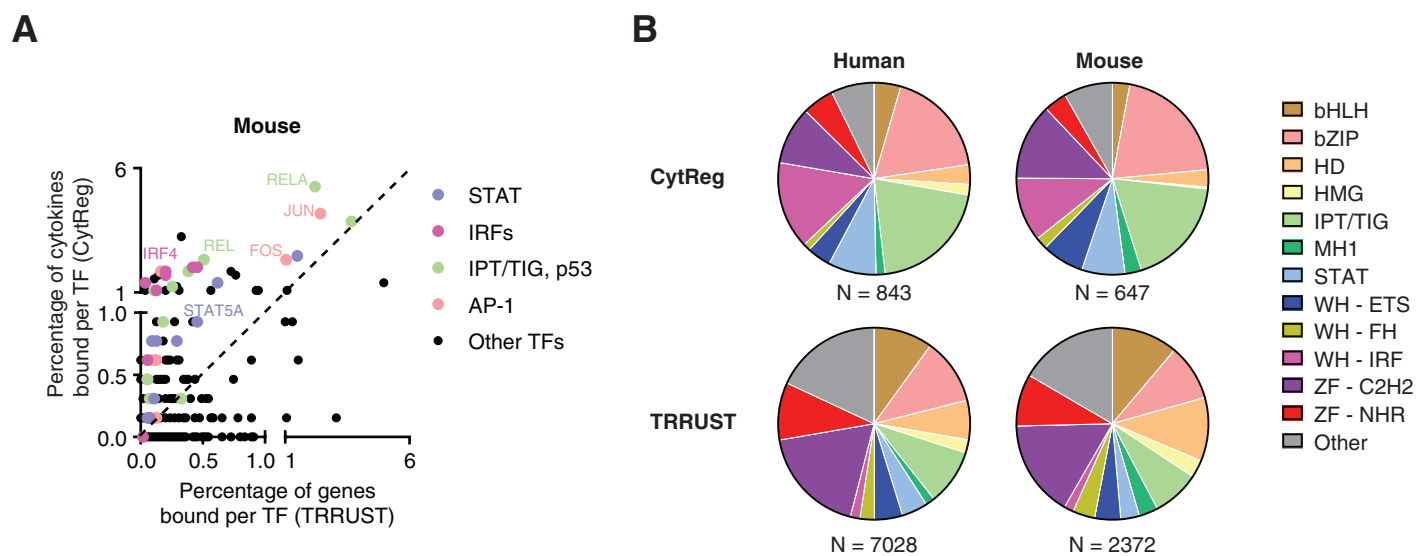

TRRUST + InnateDB

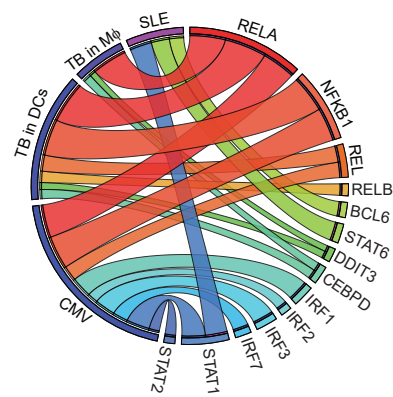

CytReg

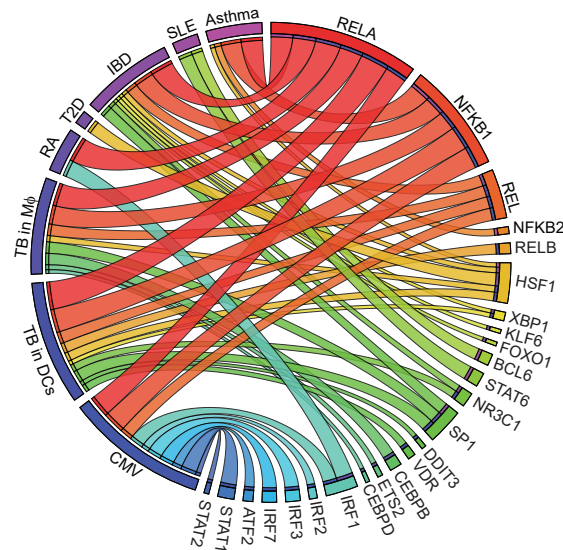

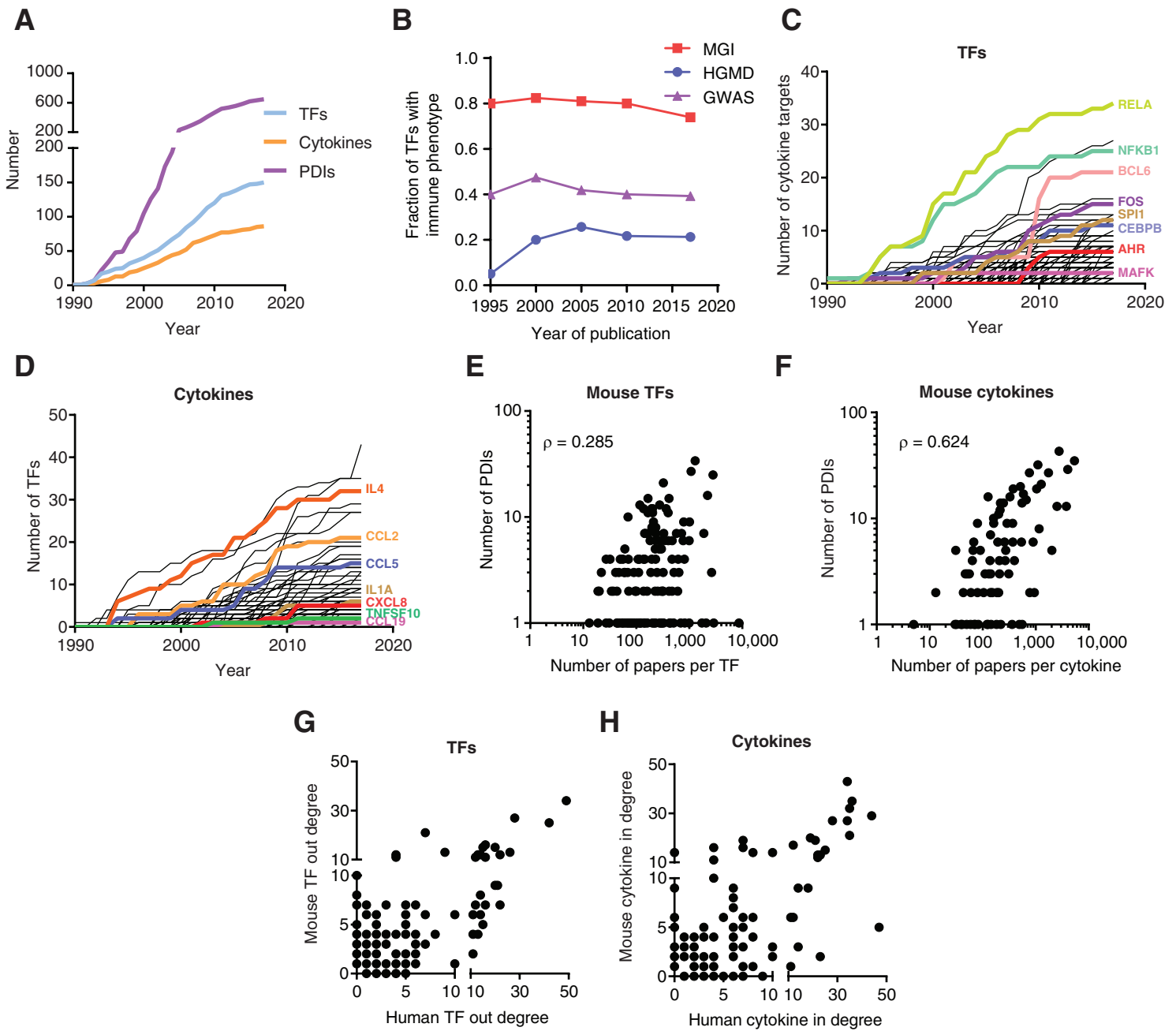

Supplement: Supplementary Data [file gky787_supplemental_files.zip › Supplementary Figures.pdf]
